# Non-Invasive Positive Pressure Ventilation for Pre-Oxygenation of Critically Ill Patients Before Intubation

**DOI:** 10.3390/jcm14155356

**Published:** 2025-07-29

**Authors:** Luigi La Via, Giuseppe Cuttone, Tarek Senussi Testa, Gilberto Duarte-Medrano, Natalia Nuno-Lambarri, Cristian Deana, Antonino Maniaci, Daniele Salvatore Paternò, Ivana Zdravkovic, Massimiliano Sorbello

**Affiliations:** 1 Department of Anesthesia and Intensive Care 1, University Hospital Policlinico “G. Rodolico—San Marco”, 95123 Catania, Italy; 2 Trauma Center Unit, “Villa Sofia-Cervello” Hospital, 90146 Palermo, Italy; giuseppe.cuttone@hotmail.it; 3 Department of Cardiac Anesthesia and Intensive Care, Cardiovascular Network, IRCCS Policlinico San Martino Hospital, 16121 Genova, Italy; t.senussi@hotmail.it; 4 Anesthesiology Department, Medica Sur Clinic & Foundation, Mexico City 11510, Mexico; dr.gilbertoduartem@gmail.com; 5 Translational Research Unit, Medica Sur Clinic & Foundation, Mexico City 11510, Mexico; nlambarri@gmail.com; 6 Surgery Department, Faculty of Medicine, The National Autonomous University of Mexico (UNAM), Mexico City 04360, Mexico; 7 Anesthesia and Intensive Care 1, Department of Emergency, Academic Hospital “S. M. della Misericordia”, Health Integrated Agency of Friuli Centrale, 33100 Udine, Italy; deana.cristian@gmail.com; 8 School of Medicine and Surgery, “Kore” University, 94100 Enna, Italy; antonino.maniaci@unikore.it (A.M.); massimiliano.sorbello@unikore.it (M.S.); 9 Department of Anesthesia and Intensive Care, Giovanni Paolo II Hospital, 97100 Ragusa, Italy; paternomd@icloud.com; 10 Anesthesia and Intensive Care, Casa di Cura Gibiino, 95100 Catania, Italy; ivana.zdravkovic82@gmail.com

**Keywords:** non-invasive positive pressure ventilation, pre-oxygenation, intubation, critical care

## Abstract

Pre-oxygenation is the key step prior to endotracheal intubation, particularly in a critically ill patient, to prevent life-threatening peri-procedural hypoxemia. This narrative review explores the emerging interest of Non-Invasive Positive Pressure Ventilation (NIPPV) as a pre-oxygenation modality in the intensive care unit (ICU) context. We reviewed data from randomized controlled trials (RCTs) and observational studies published from 2000 to 2024 that compare NIPPV to conventional oxygen therapy and High Flow Nasal Cannula Oxygen (HFNCO). The pathophysiological mechanisms for the successful use of NIPPV, including alveolar recruitment, the decrease of shunting, and the maintenance of functional residual capacity, were reviewed in depth. Existing studies show that NIPPV significantly prolongs the apnea time, reduces the rate of peri-intubation severe hypoxaemia in selected patients and is especially effective for patients with acute hypoxaemic respiratory failure. Nevertheless, appropriate patient selection is still crucial because some diseases can contraindicate or even be harmful with NIPPV. We further discussed the practical aspects of how to use this ventilatory support (the best ventilator settings, which interface, and when to apply it). We lastly discuss unanswered questions and offer suggestions and opportunities for future exploration in guiding the role of NIPPV use in the pre-oxygenation of the critically ill patient requiring emergent airway management.

## 1. Introduction

Endotracheal intubation is a high-risk procedure in critically ill patients, with complications occurring in up to 40% of cases [1]. Among these complications, cardiovascular collapse and severe hypoxemia represent the most life-threatening events, associated with cardiac arrest, neurological damage, and increased mortality [2,3]. Pre-oxygenation before intubation aims to extend the safe apnea time by maximizing oxygen reserves, primarily within the functional residual capacity, thus providing a critical safety buffer during the apneic period of airway instrumentation [4]. Conventional pre-oxygenation techniques using a face mask with 100% oxygen via a Mapleson circuit have been the standard of care for decades [5]. However, these approaches often prove inadequate in critically ill patients due to ventilation-perfusion mismatch, shunt, increased oxygen consumption, and reduced functional residual capacity [6,7]. Critically ill patients, now characterized as having a “physiologically difficult airway” [8], often present with baseline hypoxemia that makes them particularly vulnerable during intubation attempts, with reported desaturation rates as high as 50% despite standard pre-oxygenation techniques [9]. Non-invasive positive pressure ventilation (NIPPV) has emerged as a promising alternative for pre-oxygenation in this challenging population. By delivering positive pressure throughout the respiratory cycle, NIPPV can recruit collapsed alveoli, reduce shunting, and maintain functional residual capacity, potentially addressing the limitations of conventional approaches [10,11]. The first significant study exploring this concept was published by Baillard et al. in 2006, demonstrating that NIPPV improved oxygenation parameters either before and after intubation compared to standard face mask oxygenation [12]. Since this landmark study, numerous investigations have assessed the efficacy of NIPPV for pre-oxygenation in various clinical scenarios. Jaber et al. reported in a multicenter randomized controlled trial that non-invasive ventilation combined with post-intubation recruitment maneuvers significantly reduced the incidence of severe hypoxemia during intubation of critically ill patients [13]. These findings were supported by a meta-analysis by Fong et al., which showed a significant reduction in peri-intubation desaturation events with NIPPV compared to conventional methods [14]. The diffusion of high-flow nasal cannula oxygen (HFNCO) therapy has further complicated the pre-oxygenation landscape. Unlike NIPPV, HFNCO may allow continuous oxygenation during laryngoscopy, potentially offering advantages through apneic oxygenation [15,16,17]. Comparisons between NIPPV and HFNCO have yielded conflicting results, with some studies favoring NIPPV for patients with severe baseline hypoxemia [18], while others suggest comparable efficacy with potentially better tolerance for HFNCO [19]. The decisional crossroad is probably the residual amount of available functional residual capacity. Patient selection appears crucial when considering the modality of pre-oxygenation.

Certain patient populations, such as those with hypercapnic respiratory failure or acute respiratory distress syndrome (ARDS), may benefit more substantially from NIPPV [6,20]. Conversely, NIPPV may be less effective or even counterproductive in patients with hemodynamic instability, altered mental status, or upper airway obstruction [21]. The technical aspects of NIPPV delivery—including interface selection, pressure settings, and duration of application—vary considerably across studies, complicating the interpretation of results and establishment of standardized protocols [22]. Furthermore, the potential adverse effects of NIPPV, such as gastric insufflation, aspiration risk, and hemodynamic compromise, must be carefully weighed against its benefits [22,23].

The focus of this narrative review is to critically discuss ill patients who require intubation and address whether pre-oxygenation with NIPPV offers a reliable alternative to the standard approach. We will discuss the physiologic basis, clinical results, practical considerations, pitfalls, and caveats of such a strategy and curate the available evidence in this rapidly emerging field and the future of research.

## 2. Methods

This narrative review aimed to examine the evidence for non-invasive positive pressure ventilation (NIPPV) as a pre-oxygenation modality in critically ill patients before intubation. We conducted a comprehensive literature search of PubMed, EMBASE, and Cochrane Library databases from January 2000 to March 2024. Search terms included combinations of “non-invasive ventilation,” “non-invasive positive pressure ventilation,” “NIPPV,” “NIV,” “pre-oxygenation,” “preoxygenation,” “intubation,” “critically ill,” “intensive care,” and “emergency.”

We included randomized controlled trials, observational studies, systematic reviews, and meta-analyses that investigated NIPPV for pre-oxygenation in adult critically ill patients requiring intubation. Studies comparing NIPPV to conventional oxygen therapy, high-flow nasal cannula oxygen, or combined approaches were prioritized. We excluded pediatric studies, animal studies, case reports, and non-English publications.

Two authors independently screened titles and abstracts, followed by a full-text review of potentially relevant articles. Disagreements were resolved through discussion with a third author. We extracted data on study design, patient populations, interventions, comparators, outcomes (particularly oxygenation parameters, desaturation events, and complications), and methodological quality. The quality of evidence was assessed using the Oxford Centre for Evidence-Based Medicine levels of evidence framework, with randomized controlled trials and meta-analyses given the highest priority in formulating recommendations. Studies were also evaluated for risk of bias using appropriate tools based on study design.

## 3. Physiological Basis for NIPPV in Pre-Oxygenation

During apnea following induction of anesthesia, oxygen consumption continues at approximately 250–300 mL/min in critically ill patients, while carbon dioxide production generates about 200 mL/min [24].

Oxygen reserves are primarily stored in three compartments: the lungs (specifically the functional residual capacity, FRC), blood, and tissues, with the FRC representing the largest readily available oxygen reservoir [25]. In healthy adults breathing room air, the FRC contains approximately 2–2.5 L of air, equal to 450 mL of oxygen, which may increase to about 3000 mL after adequate pre-oxygenation with 100% oxygen [26]. This oxygen reserve typically allows 8–10 min of apnea before critical desaturation occurs in healthy individuals, but this duration is dramatically shortened in critically ill patients [4].

Conventional pre-oxygenation using a face mask with 100% oxygen via a Mapleson circuit faces significant limitations in the critically ill population. These patients often present with shunt fraction as high as 20–30% due to atelectasis, pulmonary edema, or pneumonia, compared to 1–2% in healthy individuals [27]. Additionally, critically ill patients frequently exhibit increased oxygen consumption from systemic inflammation, work of breathing, and catecholamine surge [28]. Their FRC is further compromised by supine positioning, obesity, pulmonary edema, and atelectasis [29]. Together, these factors create a “perfect storm” that significantly reduces the effectiveness of standard pre-oxygenation techniques, resulting in rapid desaturation during intubation attempts [9]. NIPPV addresses these physiological limitations through several mechanisms. First, the application of positive pressure throughout the respiratory cycle—particularly positive end-expiratory pressure (PEEP)—recruits collapsed alveoli and prevents derecruitment, effectively reducing shunt fraction [30]. Studies have demonstrated that NIPPV can increase end-expiratory lung volume by 10–25% in patients with acute respiratory failure [31]. Second, NIPPV maintains and potentially increases FRC, creating a larger oxygen reservoir [32]. Third, the inspiratory pressure support component of NIPPV enhances tidal volume and alveolar ventilation, facilitating more efficient oxygen loading and carbon dioxide elimination [33]. This results in higher arterial oxygen content prior to apnea, extending the safe apnea time. The physiological advantages of NIPPV over conventional oxygen delivery methods are substantial. Unlike standard face mask oxygenation, which relies on the patient’s respiratory efforts and cannot overcome the negative effects of atelectasis, NIPPV actively recruits lung units and supports ventilation [34]. Compared to HFNCO, NIPPV typically generates higher and more consistent positive airway pressures (10–15 cmH_2_O vs. a flow-dependent 2–5 cmH_2_O with HFNCO), resulting in superior alveolar recruitment [35]. However, HFNCO has the advantage of continuing oxygenation during laryngoscopy, whereas NIPPV must be discontinued [16]. Physiologically, NIPPV also differs from apneic oxygenation techniques that maintain oxygen flow during laryngoscopy. While apneic techniques rely on the mass flow of oxygen from the pharynx to the alveoli via diffusion, NIPPV actively ventilates the lungs before apnea [36]. The combination of these approaches—NIPPV for pre-oxygenation followed by apneic oxygenation during laryngoscopy—represents a physiologically sound strategy that addresses both the pre-intubation oxygen loading and the ongoing oxygen consumption during the procedure [13].

## 4. Evidence Base for NIPPV Pre-Oxygenation

**Figure 1:**
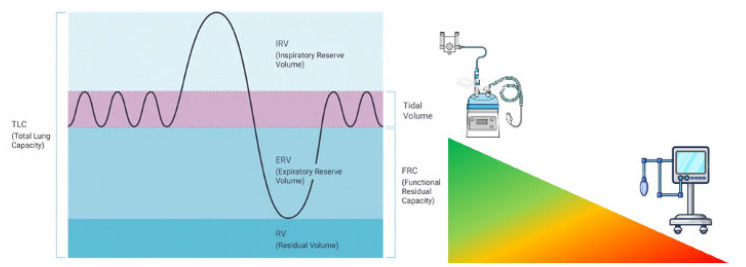
The efficacy and efficiency of the pre-oxygenation technique depend upon the amount of available functional residual capacity. The larger the reduction, the lesser the effect of non-positive pressure pre-oxygenation techniques.

The rationale for the use of NIPPV as a technique of pre-oxygenation has been fleshed out well over the last 20 years, moving from physiology to large RCTs. The idea was initially developed by observational studies concluding that PEEP had some beneficial effects on gas exchange in the critically ill. The landmark study by Baillard et al. in 2006 [12] marked the first significant clinical investigation specifically examining NIPPV for pre-oxygenation, probably standing between the first paper highlighting the difference between anatomically and physiologically difficult airways [37]. This prospective randomized study of 53 hypoxemic patients requiring intubation in the ICU compared non-invasive pressure support ventilation (PSV 5–15 cmH_2_O, PEEP 5 cmH_2_O) with standard bag-valve-mask pre-oxygenation. The results were striking: the NIPPV group achieved significantly higher mean SpO_2_ after pre-oxygenation (98 ± 2% vs. 93 ± 6%, *p* < 0.001) and maintained better oxygenation during intubation (93 ± 8% vs. 81 ± 15%, *p* < 0.001). Most importantly, the incidence of severe desaturation (SpO_2_ < 80%) decreased dramatically from 46% to 7% in the NIPPV group. This study established the proof of concept that would drive subsequent research. More interestingly, the research highlighted that arterial oxygen partial pressure remained higher during and 30 min after laryngoscopy, whereas conventional pre-oxygenation resulted in much lower values in both phases. This finding clearly suggests not only efficiency, but sustained efficacy in the peri-intubation period. Building on these findings, Vourc’h et al. [38] conducted a multicenter RCT comparing pre-oxygenation with NIPPV versus HFNCO in 124 patients with acute hypoxemic respiratory failure. While they found no significant difference in the lowest SpO_2_ during intubation between groups, a post-hoc analysis revealed a potential advantage of NIPPV in the most severely hypoxemic patients (PaO_2_/FiO_2_ < 200). This highlighted the importance of patient selection when considering NIPPV for pre-oxygenation, suggesting that HFNCO may be effective if functional residual capacity is available for being filled with oxygen; should it not be so, NIPPV has the physiologic advantage of recruiting the FRC to be later filled with oxygen [39] (Figure 1).

A methodological and logical advance came with the OPTINIV trial by Jaber et al. [13], which examined a combined approach of NIPPV pre-oxygenation with HFNCO peri-oxygenation before and during laryngoscopy. This single-center RCT of 25 patients demonstrated that the combination significantly improved the lowest SpO_2_ during intubation compared to NIPPV alone (100% vs. 96%, *p* = 0.029) and reduced the incidence of severe hypoxemia (SpO_2_ < 80%) from 21% to 0%. This study highlighted the complementary physiological benefits of combining these techniques. The FLORALI-2 study by Frat et al. [18] represented a significant advancement, directly comparing NIPPV against HFNCO with apneic oxygenation in 313 patients with acute hypoxemic respiratory failure. This multicenter RCT found that NIPPV led to better pre-oxygenation (median SpO_2_ 100% vs. 99%, *p* < 0.01) and reduced the incidence of severe desaturation below 80% (23% vs. 35%, *p* = 0.03). Notably, the benefit was most pronounced in patients with severe hypoxemia at baseline (PaO_2_/FiO_2_ < 200). Another important contribution came from Frat et al. [19], who conducted an RCT comparing HFNCO to NIPPV for pre-oxygenation in 192 critically ill patients. While they found no significant difference in the median lowest SpO_2_ during intubation (92% vs. 90%, *p* = 0.44), they observed better patient comfort with HFNCO, suggesting that factors beyond oxygenation should be considered when selecting a pre-oxygenation strategy. Several meta-analyses have synthesized this growing body of evidence. Zhao et al. [40] analyzed 11 RCTs (1472 patients) comparing NIPPV, HFNCO and conventional oxygen therapy for pre-oxygenation. They found that NIPPV significantly reduced the risk of severe desaturation compared to conventional oxygen therapy (RR 0.43, 95% CI 0.23–0.80) but found no significant difference between NIPPV and HFNCO. A network meta-analysis by Fong et al. [14] examined 25 studies (3232 patients) and concluded that NIPPV was associated with the lowest risk of desaturation events compared to all other pre-oxygenation methods. Beyond oxygenation parameters, some studies have investigated the impact of pre-oxygenation strategies on procedural outcomes. Jaber et al. [41] found that NIPPV pre-oxygenation was associated with improved first-attempt success rates compared to standard pre-oxygenation (67% vs. 54%, *p* = 0.02) in a cohort of 1000 intubations. This effect was attributed to better operator conditions due to reduced time pressure from desaturation events. Similarly, Bailly et al. [42] reported that effective pre-oxygenation was independently associated with reduced complications during intubation in a prospective observational study of 1400 ICU intubations. The most recent meta-analysis by Higgs et al. [43], including 24 RCTs (3521 patients), demonstrated that NIPPV pre-oxygenation reduced the relative risk of severe desaturation by 30% compared to conventional methods (RR 0.70, 95% CI 0.59–0.85). They also found that NIPPV was associated with reduced ICU length of stay (mean difference −1.8 days, 95% CI −3.1 to −0.5), though no significant mortality benefit was detected. This is indicative that the prevention of severe hypoxemia at intubation might have downstream effects on patient outcomes beyond the initial procedure. Although the evidence overwhelmingly supports the use of NIPPV for pre-oxygenation for most patients (especially those who are very hypoxemic), there are still questions regarding the best way to apply NIPPV, who to use it on, and the feasibility of combining approaches that would maximize the benefits while minimizing the risks.

## 5. Comparison with Alternative Techniques

Multiple pre-oxygenation strategies have recently been studied to determine optimal approaches for different clinical scenarios, and a summary of key studies is provided in Table 1.

**Figure 2:**
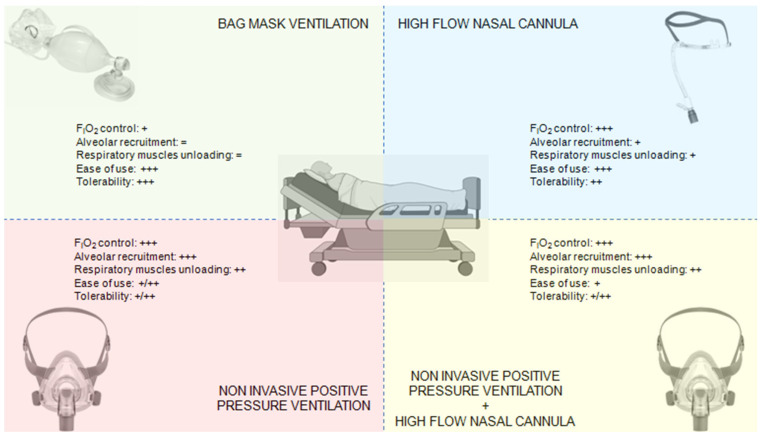
Alternative oxygenation techniques.

The primary alternatives to NIPPV for pre-oxygenation include HFNCO, standard bag-valve-mask (BVM) ventilation, and various combined approaches (Figure 2).

### 5.1. NIPPV vs. HFNCO

HFNCO has gained popularity for pre-oxygenation due to its ability to deliver high FiO_2_, provide modest positive airway pressure, and continue oxygenation during laryngoscopy [15]. Direct comparisons between NIPPV and HFNCO have yielded mixed results. The FLORALI-2 trial [18] demonstrated superiority of NIPPV over HFNCO for preventing severe hypoxemia during intubation in patients with acute hypoxemic respiratory failure. Conversely, Frat et al. [19] found no significant difference in the lowest SpO_2_ values between techniques but noted better patient comfort with HFNCO. A physiological study by Rittayamai et al. [45] showed that NIPPV achieved higher end-expiratory lung volumes and PaO2 levels compared to HFNCO, particularly in patients with more severe hypoxemia. Notably, the different mechanisms of action suggest specific clinical targets for each modality. Ricard et al. [34] observed that NIPPV was more effective in patients with recruitable lung pathology (e.g., atelectasis, early ARDS), while HFNCO offered advantages in patients with upper airway obstruction or those intolerant of masks. A pragmatic consideration highlighted by Besnier et al. [46] is that HFNCO allows continuous oxygenation during laryngoscopy, while NIPPV requires removal for tube insertion, potentially risking rapid derecruitment.

### 5.2. NIPPV vs. Bag-Valve-Mask

Traditional BVM pre-oxygenation with 100% oxygen via Mapleson circuit remains widely used but has significant limitations in critically ill patients. Baillard’s landmark study [11] demonstrated clear superiority of NIPPV over BVM, with higher post-pre-oxygenation SpO_2_ (98% vs. 93%) and reduced severe desaturation events (7% vs. 46%). A subsequent trial by Casey et al. [44] reinforced these findings, showing that BVM often fails to achieve adequate pre-oxygenation in patients with shunt physiology. The primary advantage of NIPPV over BVM lies in its ability to maintain consistent positive pressure, recruit collapsed alveoli, and provide predictable tidal volumes [47].

### 5.3. Combined Approaches

Recognition that different techniques offer complementary benefits has led to the exploration of combined approaches. The OPTINIV trial [13] demonstrated that combining NIPPV pre-oxygenation with HFNCO apneic oxygenation during laryngoscopy achieved superior results compared to NIPPV alone, effectively addressing both pre-intubation oxygen loading and ongoing oxygen consumption during the procedure. Jaber et al. reported no severe desaturation events with this combination approach. A technique gaining attention is the addition of deliberate nasal insufflation during pre-oxygenation with NIPPV (nasal oxygen during efforts securing a tube—NO DESAT), which Levitan [48] found could extend safe apnea time by up to 100 s and a recent metanalysis showed up to improve all oxygenation parameters with indirect effect on increasing intubation first-pass success [17].

### 5.4. Special Circumstances

Rapid sequence induction-intubation (RSI-I) presents unique challenges for pre-oxygenation. Traditional teaching discourages positive pressure ventilation due to aspiration concerns, potentially limiting NIPPV use. However, Baillard et al. [49] demonstrated that gentle NIPPV (pressure support ≤ 10 cmH_2_O) with proper technique minimally increases gastric insufflation while significantly improving pre-oxygenation. For RSI-I in hypoxemic patients, De Jong et al. [50] found that a modified approach using NIPPV pre-oxygenation with immediate sequence induction resulted in fewer desaturation events than conventional RSI with BVM. Nevertheless, the setting of RSII is evolving, with concerns surrounding the available evidence [51] and suggesting a change of practice, for either bag mask ventilation and cricoid force application [44]. Its practice is changing [52], with NIPPV and HFNCO already being part of the modern RSII [8] and further statements coming from the Project for Universal Management of Airways [53]. In obese patients, NIPPV shows particular advantages. Futier et al. [29] demonstrated that NIPPV pre-oxygenation with recruitment maneuvers significantly improved oxygenation and extended safe apnea time in morbidly obese surgical patients compared to conventional pre-oxygenation. This benefit extends to critically ill obese patients, where Baillard et al. [54] found NIPPV reduced atelectasis formation and improved functional residual capacity compared to standard approaches, and a recent sub-analysis of the INTUBE study confirmed these findings [1].

## 6. Patient Selection and Risk Stratification

Appropriate patient selection is crucial for maximizing the benefits of NIPPV pre-oxygenation while minimizing potential risks. The available evidence suggests that not all critically ill patients may equally benefit from this technique, calling for a careful consideration of individual patient characteristics and underlying pathophysiology.

### 6.1. Ideal Candidates for NIPPV Pre-Oxygenation

Patients most likely to benefit from NIPPV pre-oxygenation include those with acute hypoxemic respiratory failure, particularly when the PaO_2_/FiO_2_ ratio is below 200 mmHg [18]. Also, patients with atelectasis-prone conditions—including obesity, pulmonary edema, and ARDS—represent optimal candidates due to NIPPV’s ability to recruit collapsed lung units [29,55]. Cabrini et al. [56] found that patients already receiving NIPPV for respiratory support had reduced complications when continuing NIPPV during pre-oxygenation compared to transitioning to other methods.

### 6.2. Contraindications and Cautions

Despite its benefits, NIPPV pre-oxygenation is not appropriate for all patients. Absolute contraindications include inability to protect the airway, recent upper airway or esophageal surgery, undrained pneumothorax, and hemodynamic instability requiring imminent vasopressor support [57]. Relative contraindications include severe agitation, excessive secretions, facial trauma, and high aspiration risk [21]. Mosier et al. [28] cautioned that NIPPV in patients with decreased level of consciousness (GCS < 10) might increase aspiration risk, though Baillard et al. [49] demonstrated that careful application in selected obtunded patients did not increase complications.

### 6.3. Risk-Benefit Assessment in Different Patient Populations

The risk-benefit profile varies significantly across patient populations. In hypercapnic respiratory failure (e.g., COPD exacerbation), Scala et al. [58] demonstrated that NIPPV pre-oxygenation significantly reduced both desaturation events and post-intubation hypercapnia compared to conventional methods. For cardiogenic pulmonary edema, Baillard et al. [59] found NIPPV particularly effective due to its ability to simultaneously reduce preload and afterload while improving oxygenation. Conversely, in patients with COVID-19-related ARDS, Grieco et al. [33] and a recent expert consensus [60] observed that while NIPPV improved pre-oxygenation parameters, it also increased aerosolization risk, suggesting that benefits must be weighed against infection control considerations. In trauma patients, Bauman et al. [61] reported that NIPPV pre-oxygenation was beneficial only in the absence of significant chest or abdominal injuries, as positive pressure could exacerbate existing pathology. For patients with altered mental status, such as those with encephalopathy, low Glasgow Coma Scale scores, or intoxication, the application of NIPPV requires careful consideration. However, when applied with vigilant monitoring and appropriate positioning, short-duration NIPPV pre-oxygenation could still improve oxygenation parameters without increasing complications in selected patients with mild to moderate altered mental status [58].

### 6.4. Predictors of Success/Failure

Several factors predict successful NIPPV pre-oxygenation. Nava et al. [21] identified that patients who tolerate the interface well and demonstrate rapid improvement in SpO_2_ within the first 5 min are likely to achieve optimal pre-oxygenation. Jaber et al. [41] found that patients with a respiratory rate < 30 breaths/min and without accessory muscle use were more likely to benefit from NIPPV pre-oxygenation. Conversely, predictors of failure include agitation requiring sedation, inability to synchronize with the ventilator, and persistent hypoxemia despite optimal settings [62]. De Jong et al. [63] developed a risk score incorporating these factors along with BMI > 35 kg/m^2^, MACOCHA score > 3, and presence of upper airway secretions to identify patients at high risk for NIPPV pre-oxygenation failure. This score demonstrated good predictive accuracy (AUROC 0.81) and can guide clinician decision-making regarding pre-oxygenation strategy selection. Systematic evaluation of these factors allows for an individualized approach to pre-oxygenation, potentially optimizing outcomes in this high-risk procedure across diverse critically ill populations.

## 7. Practical Implementation

Successful implementation of NIPPV for pre-oxygenation requires attention to technical details, appropriate ventilator settings, careful interface selection, diligent monitoring, and smooth transition to laryngoscopy. These practical aspects can significantly impact the effectiveness and safety of the procedure.

### 7.1. Technical Aspects of NIPPV Delivery for Pre-Oxygenation

NIPPV for pre-oxygenation can be delivered through dedicated non-invasive ventilators or ICU ventilators with non-invasive capabilities (flow generators providing adequate flows in response to patients’ peak demands). Ehrmann et al. [64] demonstrated that ICU ventilators with dedicated NIV algorithms provided more stable pressure delivery and better patient-ventilator synchrony compared to basic transport ventilators. The choice of ventilation mode is also important, with pressure support ventilation (PSV) with PEEP being most commonly utilized [12,13]. Bilevel positive airway pressure (BiPAP) offers similar benefits with a potential contribution to aiding tidal volume exchanges, while continuous positive airway pressure (CPAP) alone may be insufficient for patients with high work of breathing [65].

### 7.2. Optimal Settings

The literature suggests specific ventilator settings to optimize pre-oxygenation while minimizing complications. For pressure levels, most studies recommend an inspiratory positive airway pressure (IPAP) or pressure support of 8–15 cmH_2_O and PEEP of 5–10 cmH_2_O [18]. Jaber et al. [13] demonstrated that higher PEEP levels (8–10 cmH_2_O) were particularly beneficial in severely hypoxemic patients (PaO_2_/FiO_2_ < 200). Regarding FiO_2_, Mort et al. [66] confirmed that 100% oxygen should be used to maximize oxygen storage capacity, though De Jong et al. [67] cautioned that prolonged exposure to high FiO_2_ might contribute to absorption atelectasis in some patients. The optimal duration of NIPPV pre-oxygenation remains debated. While traditional teaching recommends 3–5 min of pre-oxygenation, Tanoubi et al. [5] demonstrated that critically ill patients often require longer periods (5–8 min) to achieve maximal end-tidal oxygen concentration. A pragmatic approach suggested by Bailly et al. [42] is to continue NIPPV until end-tidal oxygen concentration reaches a plateau (typically > 90%) or for a minimum of 3 min in urgent situations.

### 7.3. Interface Selection Considerations

Interface selection significantly impacts pre-oxygenation effectiveness. Chaudhuri et al. [68] compared four interfaces (face mask, nasal mask, helmet, and total face mask) for NIPPV pre-oxygenation, finding that oronasal face masks provided the best balance of effectiveness and tolerance. Helmets, while reducing air leakage, introduced dead space that delayed oxygen equilibration. For patients with difficult facial anatomy or a beard, Chiumello et al. [69] demonstrated that total face masks achieved better seal and higher oxygen concentrations. Regardless of interface, proper sizing and fitting are essential to minimize leaks that may compromise pressure delivery and oxygenation and jeopardize patients’ compliance and adherence [70].

### 7.4. Monitoring During Pre-Oxygenation

Comprehensive monitoring is crucial during NIPPV pre-oxygenation. Beyond standard pulse oximetry and capnography, Mosier et al. [71] and Nimmagadda et Al [4] recommended monitoring expired oxygen concentration as the most reliable indicator of adequate pre-oxygenation, while Jaber et al. [41] highlighted that vigilant hemodynamic monitoring can detect early signs of positive pressure-induced hypotension, especially in volume-depleted patients.

### 7.5. Transitioning from Pre-Oxygenation to Laryngoscopy

The transition from NIPPV to laryngoscopy represents a critical moment where derecruitment and desaturation may occur. Jaber et al. [13] described a coordinated approach where NIPPV is maintained until immediately before laryngoscopy, with rapid sequence induction medications administered while NIPPV continues. The OPTINIV technique maintains nasal oxygen insufflation during this transition, providing apneic oxygenation through a separate nasal cannula [13]. For patients at high risk of desaturation, Lapinsky [72] recommended a head-up position during both pre-oxygenation and laryngoscopy to optimize functional residual capacity and reduce closing capacity, highlighting the importance of adequate positioning as a fundamental part of an adequate pre-oxygenation strategy, especially in patients with reduced FRC, such as those with obesity [73].

A standard low-flow nasal cannula may be applied during NIPPV, with the double aim of compensating for eventual leaks and being already in position for the apnoic phase of airway instrumentation [74].

## 8. Limitations and Potential Complications

Despite its benefits, NIPPV for pre-oxygenation is associated with several limitations and potential complications that must be carefully considered when selecting this technique for critically ill patients requiring intubation.

### 8.1. Potential Adverse Effects of NIPPV for Pre-Oxygenation

NIPPV can induce patient discomfort and anxiety, potentially increasing the need for sedation prior to intubation. Schmidt et al. [75] reported that approximately 30% of patients exhibited poor tolerance to NIPPV pre-oxygenation, necessitating either interface adjustment or sedative administration. Mask-related complications, including pressure ulcers, conjunctival irritation, and claustrophobia, have been documented by Carron et al. [76], though these are less concerning for short-term pre-oxygenation applications than for prolonged NIPPV use. More seriously, Lemiale et al. [77] observed that NIPPV pre-oxygenation in immunocompromised patients occasionally exacerbated respiratory distress through patient-ventilator asynchrony, particularly when inadequate support levels were used.

### 8.2. Gastric Insufflation and Aspiration Risk

One of the primary concerns with NIPPV pre-oxygenation is the potential for gastric insufflation, which may increase regurgitation and aspiration risk. Frat et al. [78] documented that inspiratory pressures exceeding 20 cmH_2_O significantly increased gastric air volume measured by ultrasound. In patients with high aspiration risk, Jaber et al. [49] recommended using lower pressure settings (PS ≤ 8 cmH_2_O, PEEP ≤ 5 cmH_2_O) and ensuring proper positioning.

### 8.3. Hemodynamic Effects

The positive intrathoracic pressure generated by NIPPV can have significant hemodynamic consequences. Ricard et al. [79] demonstrated that NIPPV reduced venous return and cardiac preload, potentially precipitating hypotension in volume-depleted patients [80]. This effect is particularly pronounced in patients with right ventricular dysfunction or pulmonary hypertension, where Mosier et al. [28] observed that NIPPV occasionally worsened right heart failure. Using a conventional medication regimen during intubation of critically ill patients, a sub-analysis of the INTUBE study clearly showed the role of propofol in causing cardiovascular collapse; no quality data are available with respect to the assumption that NIPPV may further enhance this phenomenon because of well-known effects on venous return and cardiac performance [81].

### 8.4. Delays in Securing Definitive Airway

Attempting NIPPV pre-oxygenation in inappropriate candidates may delay definitive airway management. Thille et al. [62] reported that failed NIPPV trials before intubation were associated with longer time to intubation and increased mortality in acute respiratory failure. Additionally, Kang et al. [82] observed that clinicians sometimes persisted with ineffective NIPPV pre-oxygenation despite minimal improvement in oxygenation, potentially missing the optimal window for intubation.

### 8.5. Resource Considerations

Implementation of NIPPV pre-oxygenation requires appropriate equipment and expertise. Ehrmann et al. [83] noted that dedicated NIPPV ventilators or ICU ventilators with NIV capabilities are not universally available, particularly in resource-limited settings. Furthermore, effective application requires staff familiar with NIPPV setup and troubleshooting, which may be challenging during emergent situations or off-hours when experienced personnel may be limited.

## 9. Standardized Protocols and Training for Safe NIPPV Implementation

The successful implementation of NIPPV for pre-oxygenation relies not only on equipment availability but also on the proficiency of the clinical team. Despite robust evidence supporting its physiological benefits and clinical efficacy, considerable variability persists in how NIPPV is applied across institutions. This inconsistency underscores the urgent need for standardized protocols that delineate optimal ventilator settings, interface selection, duration of application, and monitoring parameters during the pre-oxygenation phase.

Equally important is the structured training of healthcare providers involved in airway management. Anesthesiologists, intensivists, respiratory therapists, and nursing staff must receive dedicated education not only on the technical aspects of NIPPV use but also on patient selection, troubleshooting, and real-time decision-making. Simulation-based learning and interprofessional training modules can be particularly effective in promoting confidence and competence. Establishing such education as a routine component of airway management curricula may significantly enhance patient safety and procedural outcomes in high-acuity settings.

## 10. Future Directions: The Role of Artificial Intelligence

The integration of artificial intelligence (AI) into anesthesiology is poised to revolutionize perioperative care by enhancing real-time monitoring and decision-making processes. AI algorithms can analyze complex physiological data streams to predict adverse events, such as hypotension or respiratory distress, guiding airway management and allowing for preemptive interventions [84,85]. Similarly, AI-driven systems are being developed to optimize ventilator settings by continuously assessing patient-ventilator interactions, thereby reducing the incidence of asynchrony and improving overall ventilation strategies [86].

In the context of non-invasive ventilation, AI’s role extends to remote patient monitoring and tele-supervision, which are particularly beneficial in resource-limited settings or during pandemics. Advanced AI models can process data from wearable sensors and home monitoring devices to detect early signs of respiratory compromise, facilitating timely medical responses [87]. Moreover, the development of intelligent intensive care units (ICUs) equipped with AI technologies allows for continuous, autonomous monitoring of patients, enhancing the detection of subtle clinical changes that might precede deterioration. These innovations not only augment the capabilities of anesthesiologists but also contribute to more personalized and responsive patient care.

## 11. Conclusions

Non-invasive positive pressure ventilation can be a useful modality of pre-oxygenation in suitable patients with critical care-related indications for intubation. There is evidence that NIPPV may correct the physiologic deficiencies of traditional pre-oxygenation methods by opening collapsed alveoli, sustaining FRC, and optimizing ventilation-perfusion relationships. These changes lead to clinically important results that have implications for patient care, such as improved oxygen saturation during pre-oxygenation, a lower rate of high desaturation events, and possibly less intubation-related trauma. Ideally, the use of NIPPV pre-oxygenation should be placed in the management algorithm, and used in those with severe hypoxemia (i.e., PaO_2_/FiO_2_ < 200), recruitable lung pathology or with a patient who is already receiving NIPPV for respiratory support. The application should emphasize appropriate patient interface choice, determine optimal pressure values and a continuous reassessment of patient response. The combination of NIPPV pre-oxygenation and apneic oxygenation during laryngoscopy adds to established pre-oxygenation benefits and is likely to mitigate the risk of desaturation. Although not for everyone, NIPPV pre-oxygenation has carved out a place in the arsenal of tools available for airway management in the critically ill. When used appropriately, based on an understanding of the potential advantages and disadvantages, it may be an essential tool to increase the safety of this high-risk procedure and to improve patient outcomes in the intensive care unit.

## Figures and Tables

**Table 1:** Summary of Key Studies on NIPPV for Pre-oxygenation in Critically Ill Patients. NIPPV = Non-Invasive Positive Pressure Ventilation; HFNCO = High-Flow Nasal Cannula Oxygen; RCT = Randomized Controlled Trial; PSV = Pressure Support Ventilation; PEEP = Positive End-Expiratory Pressure; SpO_2_ = Peripheral Oxygen Saturation; ICU = Intensive Care Unit; RR = Relative Risk; CI = Confidence Interval.

Study	Design	Population	Intervention	Comparator	Primary Outcomes	Key Findings
Baillard et al. (2006) [12]	Prospective RCT	53 hypoxemic ICU patients requiring intubation	NIPPV (PSV 5–15 cmH_2_O, PEEP 5 cmH_2_O)	Standard bag-valve-mask	SpO_2_ after pre-oxygenation and during intubation	NIPPV group: higher mean SpO_2_ after pre-oxygenation (98 ± 2% vs. 93 ± 6%, *p* < 0.001); better oxygenation during intubation (93 ± 8% vs. 81 ± 15%, *p* < 0.001); lower incidence of severe desaturation (7% vs. 46%)
Jaber et al. (2016) [13] OPTINIV trial	Single-center RCT	25 hypoxemic ICU patients	NIPPV + HFNCO during laryngoscopy	NIPPV alone	Lowest SpO_2_ during intubation	Combined approach: higher lowest SpO_2_ (100% vs. 96%, *p* = 0.029); reduced incidence of severe hypoxemia (0% vs. 21%)
Frat et al. (2019) [18] FLORALI-2 trial	Multicenter RCT	313 patients with acute hypoxemic respiratory failure	NIPPV	HFNCO with apneic oxygenation	Lowest SpO_2_ during intubation; incidence of severe desaturation	NIPPV: better pre-oxygenation (median SpO_2_ 100% vs. 99%, *p* < 0.01); reduced incidence of SpO_2_ < 80% (23% vs. 35%, *p* = 0.03); most beneficial in severe hypoxemia (PaO_2_/FiO_2_ < 200)
Frat et al. (2019) [19]	RCT	192 critically ill patients	NIPPV	HFNCO	Median lowest SpO_2_ during intubation	No significant difference in lowest SpO_2_ (90% vs. 92%, *p* = 0.44); better patient comfort with HFNC
Vourc’h et al. (2015) [38]	Multicenter RCT	124 patients with acute hypoxemic respiratory failure	NIPPV	HFNCO	Lowest SpO_2_ during intubation	No significant overall difference; post-hoc analysis showed potential advantage of NIPPV in severely hypoxemic patients (PaO_2_/FiO_2_ < 200)
Futier et al. (2011) [29]	RCT	Morbidly obese surgical patients	NIPPV with recruitment maneuvers	Conventional pre-oxygenation	Oxygenation parameters; safe apnea time	NIPPV significantly improved oxygenation and extended safe apnea time in obese patients
Casey et al. (2019) [44]	Multicenter RCT	401 critically ill patients	Bag-mask ventilation	No ventilation between induction and laryngoscopy	Lowest SpO_2_ during intubation	Bag-mask ventilation: higher lowest SpO_2_ (96% vs. 93%, *p* = 0.01); reduced incidence of severe hypoxemia (10.9% vs. 22.8%, *p* = 0.01)
Fong et al. (2019) [14]	Network meta-analysis	25 studies (3232 patients)	Various pre-oxygenation methods	Multiple comparisons	Risk of desaturation events	NIPPV is associated with the lowest risk of desaturation events compared to all other pre-oxygenation methods
Higgs et al. (2018) [43]	Meta-analysis	24 RCTs (3521 patients)	NIPPV	Conventional methods	Severe desaturation; ICU length of stay	NIPPV reduced the relative risk of severe desaturation by 30% (RR 0.70, 95% CI 0.59–0.85); reduced ICU length of stay (mean difference −1.8 days, 95% CI −3.1 to −0.5)
Zhao et al. (2017) [40]	Meta-analysis	11 RCTs (1472 patients)	NIPPV, HFNCO, conventional oxygen therapy	Multiple comparisons	Risk of severe desaturation	NIPPV significantly reduced risk vs. conventional oxygen (RR 0.43, 95% CI 0.23–0.80); no significant difference between NIPPV and HFNC
